# Can FIB4 and NAFLD fibrosis scores help endocrinologists refer patients with non-alcoholic fat liver disease to a hepatologist?

**DOI:** 10.1590/2359-3997000000233

**Published:** 2017-01-27

**Authors:** Rodrigo Bremer Nones, Cláudia Pontes Ivantes, Maria Lucia Alves Pedroso

**Affiliations:** 1 Hospital Nossa Senhora das Graças Curitiba PR Brasil Serviço de Gastroenterologia, Hospital Nossa Senhora das Graças, Curitiba, PR, Brasil; 2 Universidade Federal do Paraná Curitiba PR Brasil Unidade de Clínica Médica, Hospital de Clínicas, Universidade Federal do Paraná (UFPR), Curitiba, PR, Brasil

**Keywords:** NAFLD, nonalcoholic steatohepatitis, mathematical model, liver fibrosis, specialist

## Abstract

**Objective:**

The objective of this study is to evaluate the performance of mathematical models used in non-invasive diagnosis of liver fibrosis in nonalcoholic fatty liver disease (NAFLD) patients to determine when the patient needs to be referred to a hepatologist.

**Subjects and methods:**

Patients referred by endocrinologists to the liver outpatient departments in two hospitals in Curitiba, Brazil, over a 72-month period were analyzed. The results calculated using the APRI, FIB 4, FORNS and NAFLD Fibrosis Score non-invasive liver fibrosis assessment models were analyzed and compared with histological staging of this population.

**Results:**

Sixty-seven patients with NAFLD were analyzed. Forty-two of them (62.68%) were female, mean age was 54.76 (±9.63) years, mean body mass index 31.42 (±5.64) and 59 (88.05%) of the 67 cases had glucose intolerance or diabetes. A diagnosis of steatohepatitis was made in 45 (76.27%) of the 59 biopsied patients, and advanced liver fibrosis (stages 3 and 4) was diagnosed in 18 (26.86%) of the 67 patients in the study population. The FIB 4 and NAFLD Fibrosis Score models had a high negative predictive value (93.48% and 93.61%, respectively) in patients with severe liver fibrosis (stages 3 and 4).

**Conclusion:**

In conclusion, use of the FIB 4 and NAFLD Fibrosis Score models in NAFLD patients allows a diagnosis of severe liver disease to be excluded.

## INTRODUCTION

Nonalcoholic fatty liver disease (NAFLD) is of considerable interest to endocrinologists because of the high prevalence of this condition in diabetic and obese patients ( 1 ). The prevalence of NAFLD in the western population is estimated to be 30% ( 2 ), a figure similar to that reported in Brazilian epidemiologic studies ( 3 , 4 ). The condition is defined as fat deposits in the liver (hepatic steatosis) similar to those found in alcohol abusers but in patients who neither consume significant amounts of alcohol nor use other substances that are a secondary cause of steatosis ( 5 , 6 ). NAFLD is classified as simple hepatic steatosis or nonalcoholic steatohepatitis (NASH). While the former accounts for the majority of cases and has a benign course ( 2 , 7 , 8 ), the latter affects 10% of patients and is characterized by steatosis accompanied by signs of cell injury (hepatocellular ballooning) and liver inflammation. In 20% of these cases, it can progress to cirrhosis and hepatocellular carcinoma ( 9 ). Distinguishing between the two conditions is a major challenge as patients are usually asymptomatic ( 10 - 12 ) with normal liver enzyme levels, and imaging tests can fail to identify the steatosis ( 2 , 7 ). A liver biopsy is the only gold-standard diagnostic test for NASH ( 9 , 13 ). However, routine biopsies are not risk-free and occasionally cannot be performed with an adequate sample size. Moreover, interobserver agreement for evaluation of histological criteria of NASH may be low ( 14 , 15 ). In recent years, there has been a search for non-invasive diagnostic methods to assess liver damage, i.e., methods for identifying liver fibrosis that can indicate possible development of advanced liver fibrosis or even cirrhosis without the need for a liver biopsy. These include a) laboratory tests used in mathematical models or diagnostic algorithms, such as the ELF panel, FibroMeter, FibroTest, NAFLD Fibrosis Score, FIB 4, FORNS and BARD ( 16 ), and (b) imaging tests, such as elastography, which assesses the elasticity of the liver ( 17 ). Non-invasive methods allow examinations to be performed in sequence to assess the course of the disease ( 7 ). However, there is a dearth of studies investigating the performance of these methods in Brazilian NAFLD patients. This study therefore sought to evaluate the results of non-invasive laboratory tests for diagnosing liver fibrosis in patients with NAFLD.

## SUBJECTS AND METHODS

The study population consisted of patients seen at the liver outpatient departments in two hospitals in Curitiba, Brazil ( *Hospital de Clínicas da Universidade Federal do Paraná* and *Hospital Nossa Senhora das Graças* ) between March 2005 and January 2011, after referral by endocrinologists. Patients with an echographic diagnosis of hepatic steatosis who agreed to have a percutaneous liver biopsy during this period were selected. Patients with liver cirrhosis secondary to NAFLD, in whom the diagnosis was based on clinical, endoscopic and/or echographic findings and who had metabolic syndrome, were also included. Other etiologies of liver disease, including alcohol abuse, hepatitis B and C infections, autoimmune hepatitis, hereditary hemochromatosis, α-1 antitrypsin deficiency, Wilson’s disease, primary biliary cirrhosis and primary sclerosing cholangitis were excluded in all the patients in the study. It was established in direct patient interviews that none of the patients selected had a history of hepatic steatosis-inducing drug use or alcohol consumption in excess of 20 g per day. Anthropometric data (weight, height and waist circumference) were collected, and body mass index [weight in kg / (height in m)^2^] (BMI) was calculated. Overweight and obesity were defined as a BMI ≥ 25 and 30, respectively. Diagnosis of glucose intolerance and diabetes followed the American Diabetes Association criteria ( 18 ), while diagnosis of metabolic syndrome was based on the NECP ATP III guidelines ( 19 ). Of the various non-invasive models for evaluating liver fibrosis, several that could be easily performed using simple demographic and laboratory data and were part of routine patient follow-up were selected. The models used were APRI ([AST level / upper limit of normal AST] x 100 / platelet count (10^9^/L)) ( 20 ), FIB 4 (age x AST / [platelet count (10^9^/L) x (ALT)^1/2^]) ( 21 ), FORNS (7.811 – 3.131 x ln [platelet count (10^9^/L)] + 0.78 x ln [GGT] + 4.367 x ln [age] – 0.014 x [total cholesterol]) ( 22 ) and NAFLD score (-1.675 + 0.037 x age + 0.094 x BMI (kg/m^2^) + 1.13 x diabetes/glucose intolerance [yes = 1, no = 0] + 0.99 x ALT/AST – 0.013 x platelet count (10^9^/L) – 0.66 x albumin (g/dL)) ( 23 ). Values for all the models were calculated for all the patients selected, apart from those for whom laboratory data were not available. In the case of patients who did not have a biopsy, the results of laboratory tests at the time of the echographic examination were used. The liver biopsies were always examined by the same pathologist. The criterion for diagnosis of steatohepatitis was the concomitant presence of hepatic steatosis, hepatocellular ballooning and lobular inflammation. At the same time, the presence and extent of liver fibrosis were also evaluated and classified as follows: stage 1, zone 3 perisinusoidal fibrosis; stage 2, portal fibrosis in addition to stage 1; stage 3, bridging fibrosis in addition to stage 2; stage 4, cirrhosis ( 13 ). The results obtained using the non-invasive models were compared with the findings of histological staging of the study population. For each model an ROC curve was fitted, the optimal cut-off point (best sensitivity and specificity) was estimated and the area under the curve (AUROC), sensitivity and specificity were calculated with a 95% confidence interval. The negative predictive value (NPV) and positive predictive value (PPV) were calculated using the data for the prevalence of liver fibrosis in each of the groups in the study population. A significance level of p < 0.05 was used. The study protocol was approved by the Ethics Committee at the *Hospital de Clínicas* , *Universidade Federal do Paraná* .

## RESULTS

**Figure 1:**
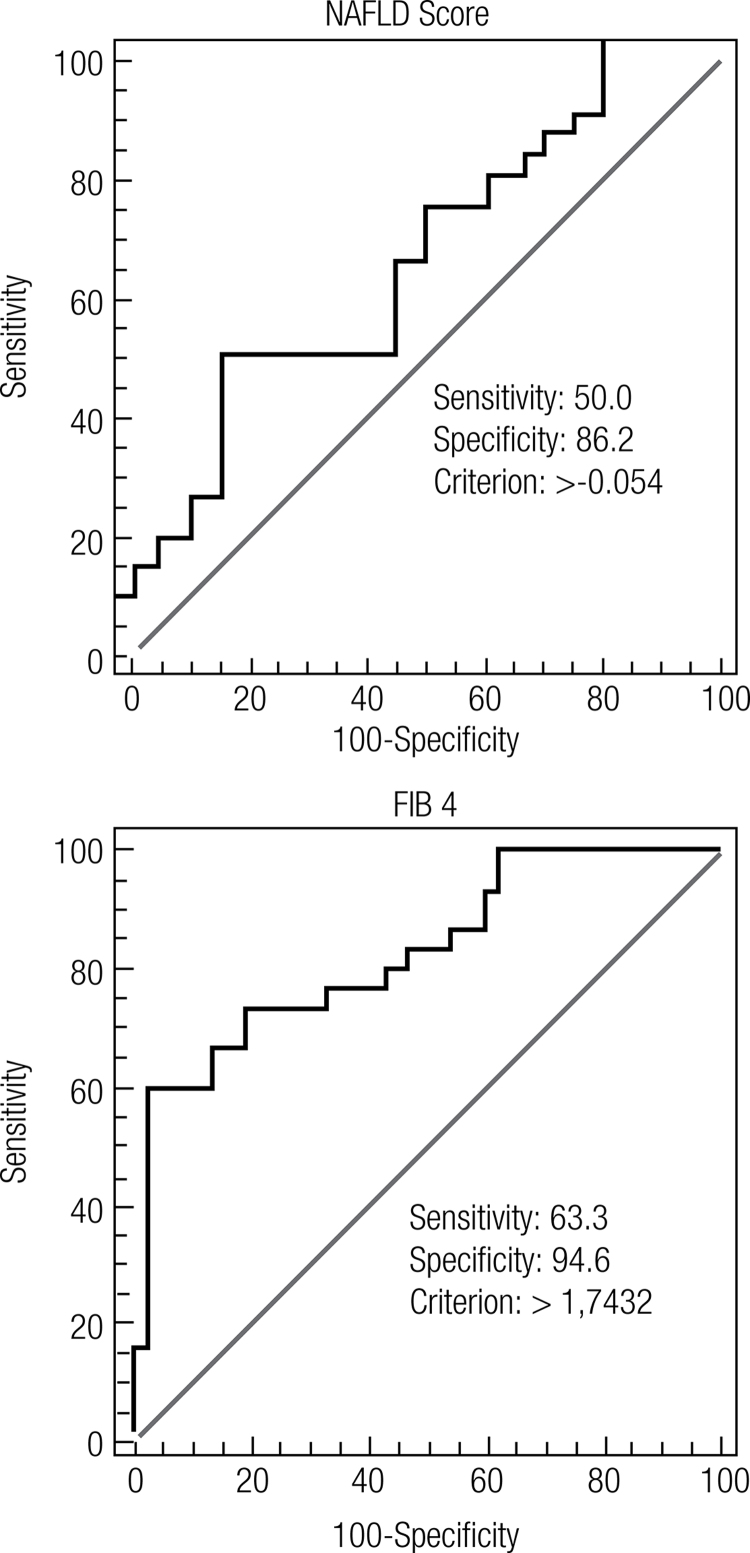
ROC curves NAFLD Score and FIB 4 models in patients in stage 2 or higher liver fibrosis.

AUROC: area under the ROC curve; PPV: positive predictive value; NPV: negative predictive value; CI: confidence interval.

In all, 195 patients were evaluated and 67 selected. Of these, 59 had a liver biopsy and 8 were diagnosed with cirrhosis based on the clinical, endoscopic and/or echographic findings. Mild or no fibrosis was present in 55% of patients, stage 2 or higher fibrosis in 45% and stage 3 or 4 fibrosis in 27%. Of the patients biopsied, 45 (76.3%) were diagnosed with steatohepatitis (p < 0.0001). Table 1 summarizes the demographic, laboratory and histological data and the results obtained using the non-invasive models to evaluate fibrosis in all the patients. The laboratory data available was used in the APRI, FIB 4, FORNS and NAFLD Score mathematical models. All the results for the non-invasive models were higher in patients with significant fibrosis. Table 2 shows the performance of each of the models for patients with stage 2 or higher liver fibrosis. The estimated AUROC as well as the cut-off points and positive and negative predictive values are also shown. Figure 1 shows the ROC curves for the non-invasive models for stage 2 or higher liver fibrosis. The best diagnostic accuracy was achieved with the FIB 4 model (AUROC = 0.83). Sensitivity and specificity varied between 50.0% and 68.2% and 79.3% and 94.6%, respectively. The best specificity was achieved with the FIB 4 model. The PPV of this model (90.47%) was also better than that of any of the other models. The NPVs for all the models were similar and varied between 68.0% and 76.1%.

**Table 1:** Demographic, laboratory and histological data and the results obtained using the non-invasive models to evaluate liver fibrosis in all the patients

	Results	Level of significance
Age (years) (n = 67)	54.76 ± 9.63	
Females (n = 67)	42 / 67 (62.68%)	p = 0.0498
BMI > 25 (n = 67)	57 / 67 (85.06%)	p < 0.0001
Waist circumference (cm) (n = 67)	102.99 ± 12.88	
**Glucose intolerance or DM (n = 67)**	**59 / 67 (88.05%)**	**p < 0.0001**
**Metabolic syndrome (n = 59)**	**46 / 59 (68.65%)**	**p = 0.0031**
Glucose (mg/dL) (n = 65)	134.02 ± 49.82	
Total cholesterol (mg/dL) (n = 59)	192.37 ± 53.31	
Platelets (10^9^/L) (n = 67)	227.94 ± 82.50	
AST (UI/L) (n = 67)	42.48 ± 30.45	
ALT (UI/L) (n = 67)	54.61 ± 41.34	
GGT (mg/dL) (n = 58)	127.74 ± 205.09	
Albumin (g/dL) (n = 56)	4.46 ± 0.61	
Size of the liver biopsy (cm) (n = 59)	2.11 ± 1.00	
Portal spaces analyzed (n = 59)	13.56 ± 5.09	
Liver fibrosis (n = 67)		
0 and 1	37 (55.22%)	
2	12 (17.91%)	
3	4 (5.97%)	
4	14 (20.89%)	
APRI (n = 67)	0.57 ± 0.54	
FIB4 (n = 67)	1.72 ± 1.43	
FORNS (n = 51)	5.17 ± 1.90	
NAFLD Score (n = 51)	-1.05 ± 1.63	

**Table 2:** Comparison of the performance of the different non-invasive models for evaluating liver fibrosis in patients with stage 2, 3 or 4 fibrosis

	APRI	FIB 4	FORNS	NAFLD Score
AUROC	0.705	0.830	0.765	0.674
95% CI	0.58-0.81	0.718-0.910	0.625-0.872	0.525-0.823
p value	0.002	0.0001	0.0001	0.035
Cut-off	0.518	1.7432	5.3097	-0.054
Sensitivity (%)	50.00	63.33	68.18	50.00
Specificity (%)	89.19	94.59	79.31	86.21
PPV	78.94	90.47	72.76	74.61
NPV	68.76	76.09	75.46	68.02

AUROC: area under the ROC curve; PPV: positive predictive value; NPV: negative predictive value; CI: confidence interval.

## DISCUSSION

In addition to being one of the main current causes of liver disease, NAFLD can be expected to be the main liver disease in the future, given the increasing prevalence of obesity and diabetes in the adult and pediatric populations ( 1 , 24 ). Diagnosis of this condition will therefore be more important for endocrinologists and for public health systems ( 6 ) as it may increase direct and indirect health costs ( 7 ), generate referrals to specialists ( 12 ) and alter morbidity and mortality of patients, due to increased risk of cardiovascular events ( 25 , 26 ), and progression of the liver disease ( 27 ). Hepatocellular carcinoma rates are expected to increase in the future ( 28 , 29 ), and NAFLD is predicted to be the main reason for liver transplants in 2020 ( 27 ). Differentiating between simple hepatic steatosis and steatohepatitis and, in particular, diagnosing the presence of significant liver fibrosis in NAFLD patients is of enormous importance for prognosis of the disease. Although liver biopsy is considered the gold standard for diagnosing and staging patients with possible NAFLD, its routine use is questionable in overweight and obese individuals, in whom the procedure may be technically more difficult and there is a higher risk of the liver fragment not being suitable for analysis ( 15 , 17 , 30 - 32 ). Furthermore, the benign progression of NAFLD in most individuals with this condition and the lack of effective treatment for steatohepatitis makes patients reluctant to undergo a biopsy ( 8 , 9 , 11 , 33 ). In addition, liver biopsies are not readily available to most of the population in Brazil, who depend on the public health service ( 34 ). These difficulties were also encountered in the present study. Assessment of the different clinical parameters analyzed in this population, such as gender, BMI, waist circumference, glucose intolerance, diabetes and metabolic syndrome, failed to identify the presence of liver fibrosis or steatohepatitis. These parameters were found in similar proportions in all the groups, as reported in other studies ( 35 ). Only patient age varied significantly, as patients with more advanced disease stage were older. This finding could be explained by NAFLD having a longer course and progressing silently with greater distortion of normal liver architecture in this population. Furthermore, the use of simple laboratory parameters, such as AST and ALT levels, did not help stage NAFLD in the patients evaluated, as has already been described in other populations and studies ( 16 , 36 - 40 ). Of the 45 patients with a histological diagnosis of steatohepatitis, 17 (37.77%) had normal levels of both transaminases, and of the 14 cirrhotic patients analyzed, four also had normal transaminase levels (28.57%). Mathematical models based on simple demographic and laboratory data are cheap, practical, easy to reproduce and allow liver fibrosis stages to be determined non-invasively in NAFLD patients. Nevertheless, in this study, these models had high NPVs in patients with advanced liver fibrosis or cirrhosis (liver fibrosis stage 3 and 4). The best NPVs were observed for the FIB 4 and NAFLD Score models (93.48% and 93.61%, respectively) using cut-off values low of 1.743 and -0.037, respectively. In other words, when these tests are carried out, 93% of cases without advanced liver fibrosis or cirrhosis would be identified, as shown in Table 3 [Table t4] . Similar performances for these two models were already reported in other populations ( 16 , 31 , 39 , 41 - 44 ) and despite their varied cut-off levels, the bulk of evidence gathered highlights the ability of these tests to indicate reliably the absence of advanced fibrosis. These indirect markers models have a high NPV, so that liver biopsies can be indicated only in cases in which there is diagnostic uncertainty about the severity of the disease. In contrast, the diagnostic performance of the models analyzed in patients with moderate or advanced liver fibrosis or cirrhosis (liver fibrosis stage 2, 3 and 4) was not uniform. The other models could not be used for this purpose as their performance was inferior. This study has shown that a greater understanding of this subject is required. Further research should therefore be undertaken with larger study populations and, if possible, the mathematical models should be used in association with other methods for non-invasive evaluation of fibrosis, such as elastography, as proposed by other authors ( 16 , 27 ). Another question that remains to be elucidated is what advantages the sequential use of these markers to monitor the progress of NAFLD in these patients may offer.

**Table 3:** NPV of the different non-invasive models for evaluation of liver fibrosis in patients with fibrosis stage 3 and 4

	Cut-off	NPV	Patients that could avoid a liver biopsy	Estimated false negative
APRI	0,4467	90,48	32/67 (52,23%)	3 (9,52%)
FIB 4	1,7432	93,48	45/67 (67,16%)	3 (6,52%)
FORNS	6,6024	89,73	37/51 (72,54%)	4 (10,27%)
NAFLD Score	-0,037	93,61	36/51 (70,58%)	2 (6,39%)

In conclusion, in this Brazilian population of NAFLD patients, referred by endocrinologists, the FIB 4 and NAFLD score mathematical models used were able to identify which patients had the greater likelihood of not having advanced fibrosis or cirrhosis. Further studies with larger populations and more cirrhotic patients should be carried out so that the findings can be compared with the results of this study.

**Table 4 t4:** NPV of the different non-invasive models for evaluation of liver fibrosis in patients with fibrosis stage 2, 3 and 4

	Cut-off	NPV	Patients that could avoid a liver biopsy	Estimated false negative
APRI	0,518	78,94	26/67 (38,80%)	14 (21,06%)
FIB 4	1,7432	90,47	22/67 (32,83%)	6 (9,53%)
FORNS	5,3097	72,76	21/51 (41,17%)	14 (27,24%)
NAFLD Score	-0,054	74,61	16/51 (31,37%)	13 (25,39%)
